# The quality and content of cerebral aneurysm related short videos on Douyin: a cross-sectional observational study

**DOI:** 10.1038/s41598-026-55531-8

**Published:** 2026-06-03

**Authors:** Guo-Ping Yang, Ke-Xin Yang, Hua-Mei Chai

**Affiliations:** 1 Department of Neurosurgery, Liangshan Yi Autonomous Prefecture Hospital of Integrated Traditional Chinese and Western Medicine, Xichang, Sichuan China; 2 Department of Obstetrics and Gynecology, The First People’s Hospital of Liangshan Yi Autonomous Prefecture, Xichang, Sichuan China

**Keywords:** Social media, Douyin, Global quality score, Modified DISCERN, Reliability, Diseases, Health care, Medical research, Neurology, Neuroscience

## Abstract

Cerebral aneurysm is a common cerebrovascular disease with a poor prognosis after rupture. This highlights the critical importance of early detection and risk factor management. Douyin has become an important channel for disseminating medical information, but the quality of video content on such platforms remains unclear. This study aims to evaluate the content, quality, and reliability of short videos related to cerebral aneurysm on Douyin. A cross-sectional analysis was conducted on 100 short videos related to cerebral aneurysm on Douyin (the Chinese version of TikTok). Video characteristics, content, uploader type, and interaction data (likes, comments, favorites, and shares) were extracted. Video quality and reliability were assessed using the Global Quality Score (GQS) and modified DISCERN (mDISCERN) tools. Videos were classified by content and emotional tone, and engagement metrics and quality scores were compared across different categories. Correlation analysis was performed between video metrics and quality scores. Videos were generally short (median: 77 s), but user engagement was high. Most videos were uploaded by cerebrovascular specialists. Educational videos scored better on GQS than case-sharing videos, but no significant difference was found in mDISCERN scores. Regarding interaction data, case-sharing videos had significantly more favorites than educational videos, but no differences were observed in other engagement metrics. Videos were further categorized by emotional tone (positive, negative, neutral), and no differences were found in quality scores or engagement metrics across these categories. Meanwhile, positive correlations were observed among engagement metrics (likes, favorites, comments, and shares). Douyin videos related to cerebral aneurysm show high engagement but slightly insufficient reliability in content. Improving video quality is essential to enhance the educational value of health information shared on the Douyin platform.

**Supplementary Information:**

The online version contains supplementary material available at 10.1038/s41598-026-55531-8.

## Introduction

Cerebral aneurysm is a pathological dilation that occurs on the wall of a cerebral artery, characterized by localized weakening and bulging of the vessel. Research indicated that approximately 3% of adults had an unruptured cerebral aneurysm, with the prevalence potentially rising to 10% in high-risk populations^1^. Once a cerebral aneurysm ruptures, it can lead to subarachnoid hemorrhage, which may result in death or severe disability. Studies showed that about 25% of patients died before reaching the hospital^2^, and even among those who were hospitalized promptly, the in-hospital mortality rate remained as high as 20%^3^. Even with timely treatment, 30% of the survivors still experienced long-term functional dependence^4^, and approximately one-third of the patients reported anxiety or depression during one-year follow-up, and with a significantly higher risk of suicide compared to the general population^5^. Current evidence suggested that smoking and hypertension were common risk factors for rupture^1,6,7^. Therefore, accurate public education is crucial to encourage at-risk individuals to seek medical care prior to rupture, enabling timely intervention and improved outcomes.

In recent years, with the diversified development of social media, a significant demographic shift has been observed in health information-seeking behavior: videos have gradually become one of the primary ways for people to access medical information, rather than traditional websites or print materials. Douyin, a popular short-video platform, boasts 1.1 billion users across more than 160 countries and regions, with 400 million daily active users in mainland China, serving as a key platform for the dissemination of public health information^8,9^. According to data recently released by Douyin, the platform’s monthly active users have officially surpassed 1 billion, making it the first short-video platform in China to reach 1 billion monthly active users. Importantly, middle-aged and older adults, who are the very population at highest risk for cerebral aneurysm rupture, now constitute a rapidly growing segment of Douyin’s user base. Consequently, Douyin has demonstrated unique influence in disseminating medical knowledge, disease education, and patient experience sharing. However, the quality of medical information on the platform varies widely, with some content lacking scientific evidence or being misleading. For example, although videos about depression and anxiety are widely circulated on Douyin, quality assessments reveal that many high-engagement videos do not provide accurate medical information^10^. Similar trends are observed in other disease areas, such as diabetes, chronic obstructive pulmonary disease (COPD), and gallstones^8,11,12^. Additionally, studies indicated that videos created by non-professionals often attract higher engagement than those by healthcare professionals, whereas the information quality was significantly lower^11,13^.

Currently, research on medical information dissemination via Douyin in the field of neuroscience remains nascent, with limited focus on cerebrovascular diseases such as cerebral aneurysm. Therefore, we retrieved 100 cerebral aneurysm-related videos from Douyin to comprehensively evaluate their interaction data—including likes, favorites, comments, and shares—and assessed video quality using the Global Quality Score (GQS) and modified DISCERN (mDISCERN) tools. Additionally, studies suggested that Douyin’s algorithm tended to recommend content with strong emotional appeal, which might affect the accuracy of medical information and audience perception^13^. Accordingly, we further categorized cerebral aneurysm related videos based on the emotional tone to assess differences in engagement and quality across emotional categories. This study not only fills the research gap in medical dissemination of cerebral aneurysms on the Douyin platform, but also provides empirical evidence for optimizing the dissemination strategies of medical knowledge on short video platforms.

## Materials and methods

### Ethical considerations

All data in this study are sourced from Douyin (a publicly accessible short video sharing platform). As the study does not involve human subject research, ethical approval is not required. Furthermore, this study does not include any personally identifiable information, ensuring privacy and confidentiality.

### Search strategy

**Fig. 1:**
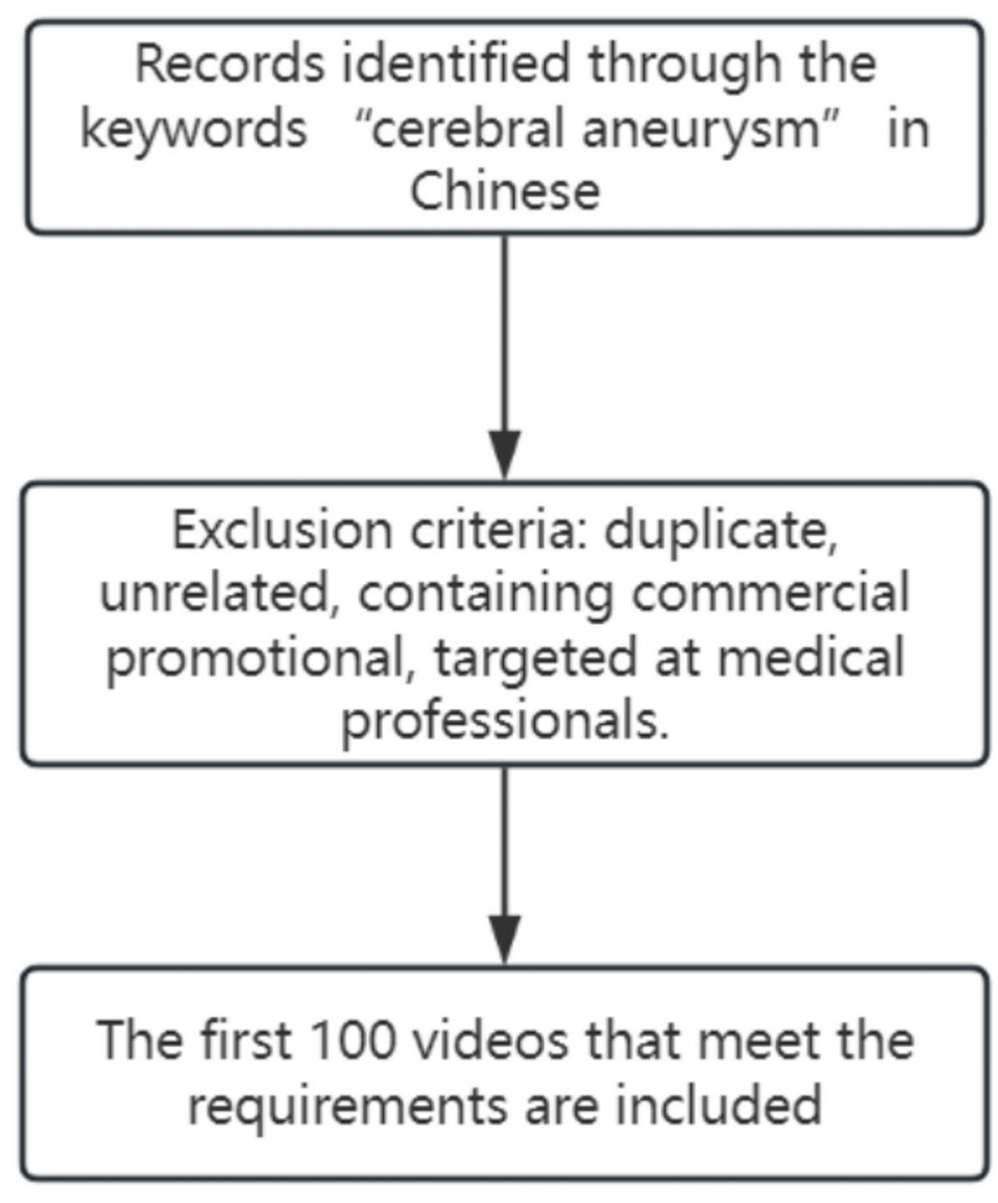
Flowchart displaying the methodology of video selection in this cross-sectional analysis.

This study employed a cross-sectional design, with all short video data sourced from Douyin (https://www.douyin.com) up to December 15, 2025. The Chinese term “脑动脉瘤” (cerebral aneurysm) was used as the search keyword. To minimize bias from personalized recommendations, the search was conducted without logging into any account, with all settings kept default. The search results were displayed in the default comprehensive ranking, and the first 100 videos meeting the following criteria were selected for subsequent analysis (Fig. 1).

Inclusion criteria: Videos related to cerebral aneurysm, including educational videos, case-sharing videos, and patient experience sharing videos.

Exclusion criteria: Duplicate videos with identical content, videos completely unrelated to the topic, videos containing commercial promotional content, and academic presentations or teaching videos targeted at medical professionals.

### Data extraction

We included video types, video uploader information, and general video information. Video types were categorized into educational videos, case-sharing videos, and patient experience sharing videos. Video uploaders were classified into three categories based on their identities: cerebrovascular specialists, including neurologists, neurosurgeons, interventional radiologists, and medical alliances formed by the aforementioned doctors; doctors from other specialties; patients and their families. General video information included the number of likes, favorites, shares, comments, and the video duration. Additionally, videos were categorized into three types based on the emotional tone: positive (videos primarily focused on alleviating patients’ disease-related concerns, such as emphasizing the treatability of the disease, clarifying that aneurysms are not cancer, etc.), negative (videos emphasized the severity of the disease, such as depicting aneurysm rupture, high risks, poor prognosis, etc.), neutral (videos with no apparent emotional bias). In subsequent analyses, we assessed whether videos with different emotional tones had different impacts on video dissemination.

**Table 1:** Characteristics of cerebral aneurysm related videos.

**Parameters **	*n (%)/M (P25, P75)*
Uploader’s type	
cerebrovascular specialists	97
doctors from other specialties	1
patients and their families	2
Video’s type	
educational videos	55
case-sharing videos	43
patient experience sharing videos	2
Video presentation format	
presenter-included	94
animation-only	6
Emotion tones	
positive	37
negative	21
neutral	42
Duration (s)	
< 60	34
60–180	64
>180	2
Duration (s)	77(53,102)
GQS score	4(3,4)
mDISCERN-1 =1	99
mDISCERN-2 =1	3
mDISCERN-3 =1	72
mDISCERN-4 =1	3
mDISCERN-5 =1	53
mDISCERN score	2(2,3)
Interaction data	
Likes	257（145,460）
Comments	26(12,73)
Favorites	50（17,131）
Shares	25(9,92)

Categorical variables are presented as frequencies and percentages; continuous variables are described using the median and the 25% and 75% quartiles.

### Quality and reliability assessment

To assess the quality and reliability of these short videos, we employed two scoring tools: the Global Quality Scale (GQS) and the modified DISCERN (mDISCERN) score.

The GQS is widely used to assess the overall quality of online videos with a five-point scale ranging from 1 to 5^14–16^. A score of 4 or 5 indicates good-to-excellent quality and flow, meaning the video is logically structured, covers most relevant information, and is useful for patients in practical terms (e.g., helping them understand disease risks, treatment options, or when to seek medical care). A score of 3 represents moderate quality, where some important information is adequately discussed but key elements may be missing. Scores of 1 or 2 indicate poor or generally poor quality, characterized by illogical content, missing information, and limited utility for patients.

DISCERN was originally designed to evaluate the reliability and quality of consumer-oriented health information, consisting of 16 items^17,18^. Subsequently, modified DISCERN was developed to meet the evaluation needs of different application scenarios. The mDISCERN score consists of five questions, with one point awarded for each question met. The higher total score indicates the greater reliability. Specifically, mDISCERN assesses whether the video is clear, concise, and understandable; whether valid sources are cited; whether the content is presented in a balanced and unbiased manner; whether additional sources of information are listed for patient reference; and whether areas of uncertainty are mentioned. Thus, a low mDISCERN score reflect deficiencies in information reliability (e.g., lack of source attribution, unbalanced presentation, or omission of uncertainties).

The two scales focus on different aspects: the GQS emphasizes whether users can benefit from the information conveyed in the video, while the mDISCERN score focuses more on the accuracy, reliability, and impartiality of the information presented, and the two are often used together to provide a more comprehensive assessment. The specific content of the two scales is provided in Supplementary Tables 11 and 2.

**Table 2:** Detailed characteristics of videos under different categories.

**Parameters**	**n** **(%)**	**Likes**	**Comments**	**Favorites**	**Shares**	**GQS score**	**mDISCERN score**
Uploader’s type							
cerebrovascular specialists	97	259 (141,480)	26 (12,73)	53 (17,138)	26 (9,94)	3 (3,4)	2 (2,3)
doctors from other specialties	1	147*	8*	15*	9*	3*	1*
patients and their families	2	244*	23*	59*	14*	3*	1*
Video presentation format							
presenter-included	94	258 (146,474)	28 (12,73)	56 (17,143)	26 (9,94)	4 (3,4)	2 (2,3)
animation-only	6	231 (83,802)	23 (7,35)	19 (4,52)	11 (5,79)	4 (3,4)	2 (2,2)
Video’s type							
educational videos	55	219 (102,396)	22 (9,66)	40 (11,100)	23 (6,71)	4 (3,4)	2 (2,3)
case-sharing videos	43	302 (177,722)	45 (16,81)	76 (29,193)	42 (11,138)	3 (3,4)	2 (2,3)
patient experience sharing videos	2	244*	23*	59*	14*	3*	1*
Emotion tones							
positive	37	323 (134,582)	42 (10,75)	87 (11,188)	25 (10,127)	3 (3,4)	2 (2,3)
negative	21	205 (140,316)	17 (12,27)	41 (26,68)	21 (8,70)	4 (3,4)	2 (2,3)
neutral	42	261 (147,522)	34 (15,75)	62 (16,125)	32 (8,81)	3 (3,4)	2 (2,3)

categorical variables are presented as frequencies and percentages;continuous variables are described using the median and the 25% and 75% quartiles.* indicates quartiles cannot be calculated, due to insufficient sample size.

In this study, the scoring was independently conducted by two neurologists. For videos with significant scoring discrepancies, a third rater resolved the disagreements and provided the final score. Ultimately, all raters reached consensus on all scores. Prior to scoring, they received uniform training to ensure consistency in evaluation criteria and minimize bias. The inter-rater reliability for quality and reliability assessments was evaluated using Cohen’s Kappa coefficientt, with values interpreted as follows: >0.8, excellent agreement; 0.6–0.8, substantial agreement; 0.4–0.6, moderate agreement; and < 0.4, poor agreement.

**Table 3:** Quality analysis of different video types.

**Parameters**	**Educational videos**	**Case-sharing videos**	**P**	**Z**
GQS	4(3,4)	3(3,4)	＜0.001	-4.317
mDISCERN-1	1(1,1)	1(1,1)	0.377	-0.884
mDISCERN-2	0(0,0)	0(0,0)	0.71	-0.372
mDISCERN-3	1(1,1)	1(0,1)	＜0.001	-3.482
mDISCERN-4	0(0,0)	0(0,0)	0.122	-1.548
mDISCERN-5	0(0,0)	1(0,1)	0.02	-2.335
mDISCERN-total	2(2,3)	2(2,3)	0.712	-0.369

Continuous variables are described using the median and the 25% and 75% quartiles.

### Statistical analysis

In this study, categorical variables were presented as frequencies and percentages, while continuous variables, as they did not meet the normality assumption, were described using the median and the 25% and 75% quartiles. The inter-rater reliability for the GQS and modified DISCERN scores was assessed using Cohen’s kappa coefficient, with a kappa value ≥ 0.8 indicating excellent agreement. The Mann-Whitney U test was employed to compare interaction data and quality scores between educational videos and case-sharing videos. For comparisons among the three groups of videos with different emotional tones—positive, negative, and neutral—the Kruskal-Wallis test was applied. Spearman correlation analysis was conducted to explore the relationships among video interaction data, GQS scores, and modified DISCERN scores. All statistical analyses were performed using IBM SPSS Statistics 25, with a significance threshold of *P* < 0.05.

**Table 4:** Differences in data for different types of videos.

**Parameters**	**Educational videos**	**Case-sharing videos**	**P**	**Z**
Likes	219(102,396)	302(177,722)	0.114	-1.582
Favorites	40(11,100)	76(29,193)	0.016	-2.41
Comments	22(9,66)	45(16,81)	0.11	-1.597
Shares	23(6,71)	42(11,138)	0.065	-1.844
Duration (s)	79(53,103)	71(52,102)	0.66	-0.44

Continuous variables are described using the median and the 25% and 75% quartiles.

## Results

### Video characteristics

**Fig. 2:**
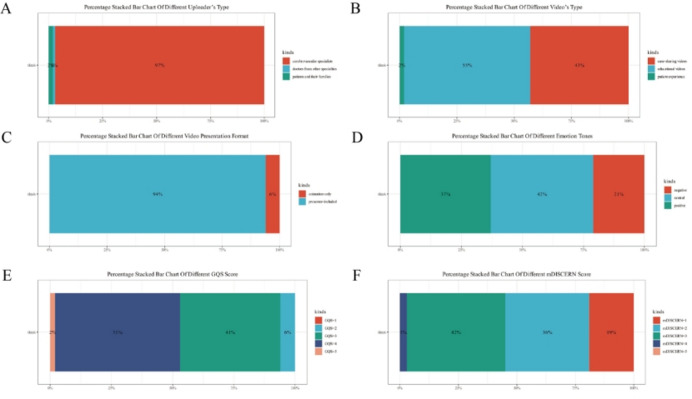
Percentage stacked bar chart of various types of videos under different video categories. (**A**) Distribution of different video uploaders in the included videos. (**B**) The distribution of different video types in the included videos. (**C**) The distribution of different video presentation formats in the included videos. (**D**) The distribution of different video emotion tones in the included videos. (**E**) The distribution of different GQS score in the included videos. (**F**) The distribution of different mDISCERN score in the included videos

This study ultimately included 100 videos after strictly applying the inclusion and exclusion criteria. As shown in Table 1, the total engagement metrics of these videos were as follows: 62,558 likes, 13,782 favorites, 14,811 comments, and 10,645 shares, with a median video duration of 77 s. Analysis of the video uploaders revealed that 97% of the videos were uploaded by cerebrovascular specialists, 1% by critical care physicians, and 2% by patients with cerebral aneurysms, indicating that Douyin had become an important platform for medical professionals to disseminate health information. In terms of video categories, 55% were related to cerebral aneurysm education, while 43% consisted of case sharing. Regarding emotional tones, 37% of the videos were positive, 21% were negative, and 42% were neutral. Under the aforementioned classification methods, the proportion of different types of videos was shown in Fig. 2.

Further classification based on video source and production characteristics was summarized in Table 2. This table presented the engagement metrics (likes, comments, favorites, and shares) and quality assessments of the videos across four dimensions: uploader type, video presentation format (presenter-included or animation-only), video type, and emotional orientation.

**Table 5:** Differences in data for videos with different emotion tone.

**Parameters**	**positive (n=37)**	**negative (n=21)**	**neutral (n=42)**	**P**
Likes	323(134,582)	205(140,316)	261(147,522)	0.501
Favorites	87(11,188)	41(26,68)	62(16,125)	0.855
Comments	42(10,75)	17(12,27)	34(15,75)	0.244
Shares	25(10,127)	21(8,70)	32(8,81)	0.835
GQS	3(3,4)	3(3,4)	3(3,4)	0.106
mDISCERN total	2(2,3)	3(2,3)	2(2,3)	0.668

Continuous variables are described using the median and the 25% and 75% quartiles.

### Video quality analysis

For the GQS score, the inter-rater reliability (Cohen’s κ) was 0.924 (*P* < 0.001), and for the mDISCERN score, the inter-rater reliability (Cohen’s κ) was 0.882 (*P* < 0.001), indicating a high level of agreement among raters.

The median GQS score for all videos was 4, suggesting that these videos provided good practical value in terms of content and effectively address viewers’ questions regarding cerebral aneurysms. However, the mDISCERN score was only 2, indicating deficiencies in the reliability of the information presented. Further analysis of the mDISCERN scores revealed that the majority of the included 100 videos were based on personal experience sharing, with only three videos citing information sources or providing further references for viewers (Table 1), which contributed to the overall low scores.

Subsequently, we further evaluated the quality scores of educational videos and case-sharing videos. The results showed that educational videos had higher GQS scores, while no statistically significant difference was observed in the total mDISCERN scores between the two categories (Table 3).

**Table 6:** Correlation analysis of video variables.

Parameters	Likes		Favorites		Comments		Shares		GQS		mDISCERN total	
	ρ	P	ρ	P	ρ	P	ρ	P	ρ	P	ρ	P
Likes	-	-	0.880	＜0.001	0.867	＜0.001	0.837	＜0.001	-0.249	0.013	-0.105	0.298
Favorites	0.880	＜0.001	-	-	0.830	＜0.001	0.927	＜0.001	-0.311	0.002	-0.119	0.237
Comments	0.867	＜0.001	0.830	＜0.001	-	-	0.824	＜0.001	-0.206	0.040	-0.024	0.816
Shares	0.837	＜0.001	0.927	＜0.001	0.824	＜0.001	-	-	-0.232	0.020	-0.085	0.402
Duration (s)	-0.027	0.787	-0.006	0.955	-0.091	0.370	0.024	0.813	0.099	0.327	-0.142	0.159
GQS	-0.249	0.013	-0.311	0.002	-0.206	0.040	-0.232	0.020	-	-	0.418	＜0.001
mDISCERN total	-0.105	0.298	-0.119	0.237	-0.024	0.816	-0.085	0.402	0.418	＜0.001	-	-
Video upload days	-0.020	0.844	-0.020	0.840	-0.027	0.792	-0.003	0.974	-0.058	0.567	0.108	0.285

### The quality and popularity of videos with different contents and different emotional tones

The videos were categorized based on their types into educational videos, case-sharing videos, and patient experience sharing videos. Among these, only two videos belonged to the patient experience sharing category. Therefore, our analysis primarily focused on comparing the differences in engagement metrics and quality assessments between educational videos and case-sharing videos.

As shown in Table 4, educational videos demonstrated higher GQS scores. In contrast, case-sharing videos exhibited better performance in engagement metrics such as likes, favorites, shares, and comments, with particularly statistically significant advantages in the number of favorites. We further analyzed these findings. Educational videos tended to provide more comprehensive information, which better addressed viewers’ questions and concerns, thereby achieving higher GQS scores. On the other hand, case-sharing videos focused on specific patients and their conditions, creating a stronger sense of relatability for viewers, which contributes to their superior engagement metrics.

Additionally, we examined the differences in engagement metrics and quality scores among videos with different emotional tones. The results, presented in Table 5, indicated no statistically significant differences in quality scores or engagement metrics among videos with positive, negative, or neutral emotional tones. This suggested that viewers’ perceptions of the videos were not substantially influenced by the emotional tone of the content.

### Correlation analysis

**Fig. 3:**
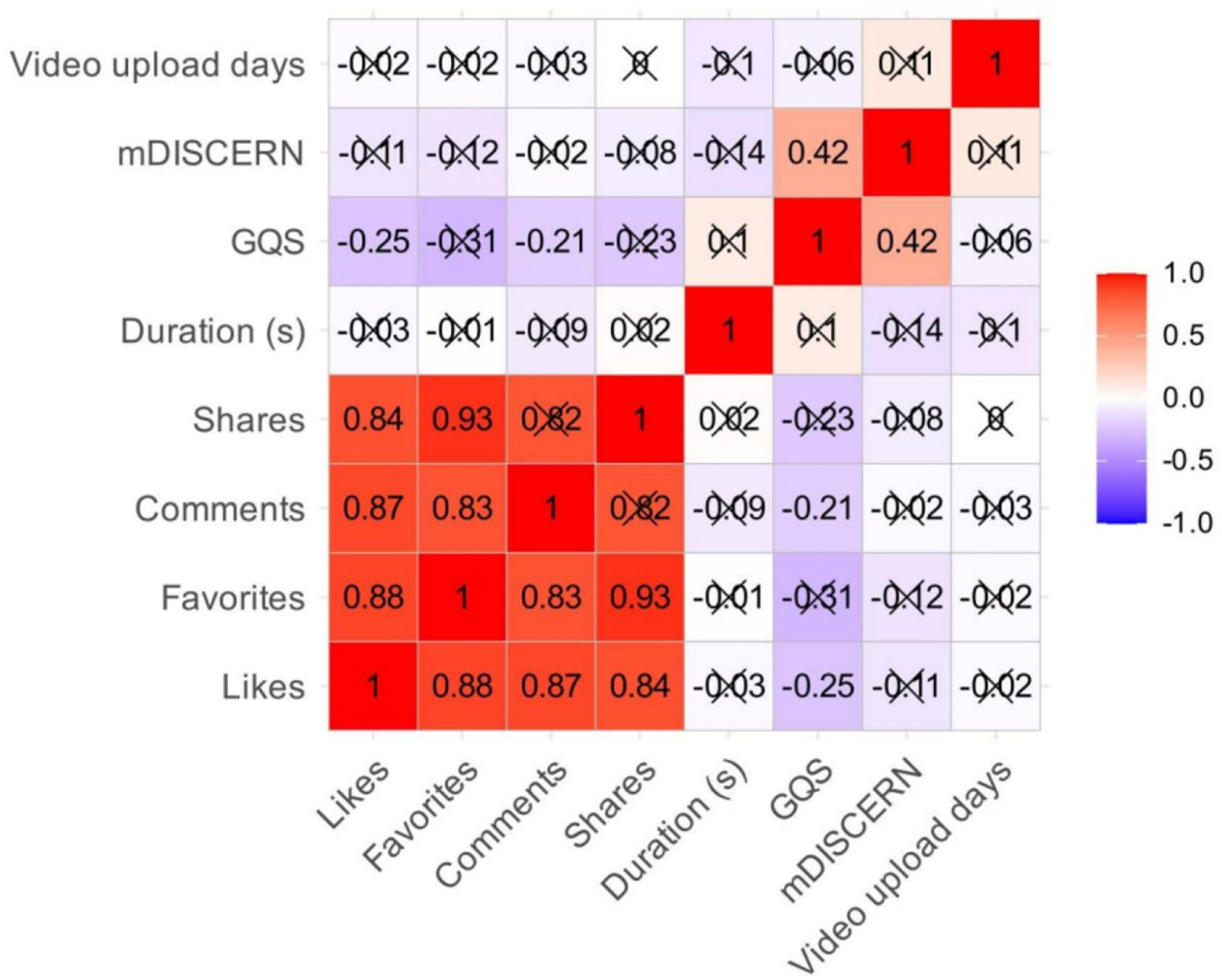
Correlation heatmap of video variables. The correlation heatmap of video variables, including likes, favorites, comments, shares, duration (s), GQS, mDISCERN, and video upload days. X represents no correlation, and the values in the graph are the correlation ρ values

Spearman’s rank correlation coefficient (ρ) analysis was used to determine the relationships among various video variables. As shown in Table 6, the results indicated that all engagement metrics—likes, comments, shares, and favorites—were positively correlated with each other (all *P* < 0.05). Additionally, all engagement metrics showed significant negative correlations with GQS scores (all *P* < 0.05), but no correlations were found with mDISCERN scores (all *P* > 0.05). A positive correlation was also observed between GQS scores and mDISCERN scores (*P* < 0.05). Video duration and the number of days since upload showed no correlations with engagement metrics or quality scores (all *P* > 0.05). The above correlation analysis results were visualized through a heatmap, as shown in Fig. 3.

## Discussion

Current research indicates that videos have become an important channel for medical professionals to disseminate medical information and for the public to access such information^19–24^. Our study evaluated the quality, content characteristics, and engagement metrics of videos related to cerebral aneurysms on Douyin. The results revealed that there were still deficiencies in the quality of these videos. By analyzing the related content, this study provided insights into how medical professionals could enhance the quality of their medical education for publics.

### Video quality assessment

To evaluate the quality and reliability of these short videos, we used two scoring tools: the GQS and the mDISCERN score. These two scales focus on different aspects: GQS emphasizes whether users can benefit from the information conveyed in videos, while mDISCERN rating focuses more on the accuracy, reliability, and fairness of the presented information. In short, GQS reflects perceived usefulness and presentation quality, whereas mDISCERN assesses source transparency and informational reliability.

The median GQS score for these videos was 4. Even when categorizing the videos by uploader type, video category, or emotional tone, the median GQS score for each group was no lower than 3. This indicated that the video content was effective in addressing viewers’ questions and meeting their needs. However, the median mDISCERN score was only 2, suggesting possible deficiencies in the credibility of the information presented in these videos. Further analysis revealed that the vast majority of videos were based on the creators’ personal clinical experiences or training, with only 3% of the videos clearly citing the sources of their data or providing references for further information. This shortcoming revealed that the need for medical professionals engaged in public medical education to ensure that data used in their content was supported by clear references, thereby improving the reliability of the information and reducing the dissemination of misinformation stemming from personal experiences.

### Correlation between emotional tones and engagement metrics

Our study found that there was no significant association between emotional tone (positive, negative, or neutral) and video engagement metrics. We acknowledge that this finding is counterintuitive and have considered several possible explanations.

First, the homogeneity of the video producer may have contributed to this finding. Nearly all videos in our study were produced by doctors, whose professional training and communication style may naturally constrain emotional expression within a relatively narrow, clinically grounded range.

Second, the relationship between emotional tone and health-related behaviors is not unidirectional. For example, negative emotions such as fear or anxiety may motivate some viewers to seek further information or take preventive action, but may also lead to defensive avoidance.

Third, engagement metrics are multidimensional (e.g., likes, favorites, comments, shares). Different viewer subgroups may contribute differently to these metrics. For instance, viewers whose personal experience aligns closely with the video content may be more likely to save the video, whereas those who perceive relevance to a friend or family member may be more inclined to share it. Unfortunately, viewer-level characteristics can not be captured from the publicly available data of the videos, precluding a more granular analysis of how emotional tone might interact with viewer traits to influence specific engagement behaviors.

### Correlation between video quality and characteristics

Our analysis showed that interaction metrics such as likes, comments, favorites, and shares were highly correlated, but not significantly correlated with mDISCERN. This result indicated that there was a mismatch between the popularity of videos and informational rigor.

### Further suggestions for disseminating medical information through video

Although most of the videos included in our study were created by medical professionals, the medical information conveyed in some of them lacked strong supporting evidence from authoritative data sources. We must acknowledge that even among professional medical personnel, the accuracy of their specialized knowledge may vary due to differences in educational backgrounds, disparities in ongoing professional training, and other factors, potentially leading to inaccuracies^25,26^. The lack of correlation between video engagement metrics and mDISCERN scores, combined with a negative correlation with GQS scores, suggested that users might be more concerned about the usefulness of video content rather than the rigor of information. Based on our findings, we offer several considerations that may help inform efforts to the quality of such videos and thereby providing the public with more accurate and effective knowledge.

First, our finding that videos often achieved high GQS scores but low mDISCERN scores highlights a specific issue: such videos are generally clear, and useful for patients, but lack academic rigor in information source attribution. To address this, creators could display source references as on-screen text during the relevant segment. Additionally, more detailed citations, such as DOIs or links to original articles, could be placed in the video description. Unverified public information or relying solely on personal experience should be avoided^27,28^. Additionally, implementing a peer-review system for medical videos could be beneficial. This system could involve preliminary assessment of content accuracy using artificial intelligence (AI) tools before publication, followed by content review by other medical professionals^29^. In this framework, AI models could be designed to synthesize and integrate the most current clinical guidelines from relevant international medical societies. The AI could then assist in reviewing and revising the content of medical videos, for example, by flagging statements that contradict established recommendations or identifying unsupported claims.

Second, when creating medical content, creators should aim to enhance the dissemination of their videos while ensuring content quality. As shown in Table 2, videos featuring actual medical professionals yielded higher engagement metrics compared to those using only animation. This suggests that the presence of medical professionals may facilitate wider dissemination of content, which is consistent with existing researches. Furthermore, as indicated in Table 3, case-sharing videos tended to foster greater viewer engagement than purely educational content. This indicates the value of incorporating real patient narratives into videos to make the content more relatable and compelling^30^.

Third, the refinement of recommendation algorithms may be helpful to enhance the dissemination of high-quality content. For example, priority in recommendations could be given to professionally reviewed, high-quality medical videos, while the promotion of lower-quality content should be reduced, and videos containing clearly erroneous information could be subject to stricter pre-upload review processes. Such measures would not only effectively improve the quality of medical information accessible to users but also incentivize medical content creators to enhance the quality of their work, thereby fostering a positive cycle of development.

### Strengths and limitations

Our study evaluated the quality and reliability of cerebral aneurysm related videos on Douyin, filling a gap in the current research. However, this study also had several limitations.

First, our sample is restricted to the top 100 videos retrieved on a single platform (Douyin), using the Chinese term for “cerebral aneurysm”. Douyin hosts millions of videos related to cerebrovascular diseases, and analyzing only 100 videos may result in insufficient coverage. Moreover, Douyin’s video recommendation algorithm operates in real time and may be updated or changed over time, which could undermine the stability of the conclusion. In addition, different short-video platforms may adopt different algorithms. Therefore, the conclusions of this study cannot be generalized to all video platforms. Further generalization of the conclusion would require large-scale, multi-platform research for support.

Second, regarding the sample size, although some previous similar studies also used the top 100 videos in the search list as the inclusion sample, we have to acknowledge that limiting the analysis to the top 100 results of a single search query may result in insufficient coverage.

Third, the widely used positive-feedback-driven recommendation algorithms might disproportionately amplify certain types of content that were likely to trigger strong emotional reactions. For example, excessive exposure to content focusing on treatment failure could deter patients from seeking necessary medical care, while overrepresentation of consistently positive outcomes might lead to an underestimation of potential risks. To minimize potential interference from personalized recommendations, our study employed non-logged-in search methods when selecting videos. However, we acknowledged that this approach could not fully replicate or assess the complex algorithmic mechanisms employed by Douyin in real-world user experiences. Consequently, any discussion about the potential impact of platform algorithms on content visibility remained inferential.

Fourth, due to the severe imbalance in the distribution of uploader types, our study only included videos uploaded by specialists in the relevant analyses. Therefore, our findings cannot reflect the perspectives of non-professionals regarding cerebral aneurysm.

Fifth, this study is descriptive and speculative. We assessed associations among different parameters but cannot determine causality. For example, while we found no significant association between emotional tone and engagement metrics, the underlying reasons cannot be evaluated from our data and can only be speculated. The possible explanations include the homogeneity of video creators, the bidirectional nature of emotion on health-related behaviors, and differences in viewer-level characteristics.

Sixth, and most importantly, this study does not provide any evidence that viewing or creating such videos improves patient outcomes, clinical decision-making, or health behaviors. So, we cannot give any clinical advice based on this study.

## Conclusion

Our study identified deficiencies in the quality of cerebral aneurysm related videos sampled from Douyin, suggesting that caution might be warranted when using such platforms for medical information seeking. However, as mentioned in the limitations section, this study is small-scale, single-platform, descriptive, and speculative. Therefore, before the conclusions can be widely generalized, larger-scale, multi-platform, and longitudinal studies are needed.

## Supplementary Information

Below is the link to the electronic supplementary material.

Supplementary Material 1

Supplementary Material 2

## Data Availability

The datasets supporting the conclusions of this article are included within the article and its additional files.
